# Occupational risk perception and its associated factors among nurses and physicians in Peruvian health facilities

**DOI:** 10.47626/1679-4435-2021-928

**Published:** 2023-11-24

**Authors:** Álvaro Nicolás Méndez-Maturrano, José Luis Perales-San-Miguel, J. Jhonnel Alarco

**Affiliations:** 1 Carrera de Medicina Humana, Universidad Científica del Sur, Lima, Perú

**Keywords:** occupational risks, occupational diseases, risk factors, physicians, nurses, Peru, riesgos laborales, enfermedades profesionales, factores de riesgo, médicos, enfermeras y enfermeros, Perú

## Abstract

**Introduction:**

A high perceived risk is related to lower rates of occupational accidents in the health personnel.

**Objectives:**

To determine the levels of occupational risk perception and its associated factors in nurses and physicians from health facilities in Peru during 2016.

**Methods:**

An analytical cross-sectional study was conducted with secondary data from the National Survey of Health Users Satisfaction (Encuesta Nacional de Satisfacción de Usuarios en Salud) 2016. The problem variable was the occupational risk perception, and sociodemographic variables and variables related to occupational risk exposure were included as possible associated factors. Crude and adjusted ordinal logistic regression models were developed to determine the associated factors. All estimates were weighted according to the National Survey of Health Users Satisfaction 2016 complex sampling.

**Results:**

Levels of perceived occupational risk were similar between nurses and physicians. Weekly working hours, having a previous work accident, and receiving protective equipment were found to be associated with occupational risk perception in nurses. Age, institution of origin, having a specialty, suffering from a chronic disease, and receiving occupational risk training were found to be associated with occupational risk perception in physicians.

**Conclusions:**

In Peru, the levels of occupational risk perception in nurses and physicians are similar. However, the associated factors differ according to the profession. These findings may contribute to the norms or laws related to the occupational safety of health personnel.

## Abstract (es)

**Introducción:**

Una alta percepción de riesgo está relacionada con menores tasas de accidentes laborales en el personal sanitario.

**Objetivos:**

Determinar los niveles de percepción de riesgo ocupacional y sus factores asociados en enfermeros y médicos de establecimientos de salud del Perú durante el 2016.

**Métodos:**

Se realizó un estudio transversal analítico de los datos secundarios de la Encuesta Nacional de Satisfacción de Usuarios en Salud 2016. La variable problema fue la percepción de riesgo ocupacional y cómo posibles factores asociados se incluyeron variables sociodemográficas y variables relacionadas con la exposición a riesgos laborales. Se elaboraron modelos crudos y ajustados de una regresión logística ordinal para determinar los factores asociados. Todos los cálculos se ponderaron según el muestreo complejo de la Encuesta Nacional de Satisfacción de Usuarios en Salud 2016.

**Resultados:**

Los niveles de percepción de riesgo ocupacional fueron semejantes entre enfermeros y médicos. Las horas de trabajo semanal, tener un accidente de trabajo previo y recibir equipos de protección resultaron ser factores asociados a la percepción de riesgo ocupacional en los enfermeros. La edad, la institución de procedencia, tener una especialidad, tener una enfermedad crónica y recibir capacitaciones en riesgo laboral resultaron ser factores asociados a la percepción de riesgo ocupacional en los médicos.

**Conclusiones:**

En el Perú, los niveles de percepción de riesgo ocupacional en enfermeros y médicos son semejantes. Sin embargo, los factores asociados difieren según la profesión. Estos hallazgos pueden contribuir con las normas o leyes relacionadas con la seguridad en el trabajo del personal sanitario.

## INTRODUCTION

The International Labor Organization (ILO) estimates that more than 2.7 million deaths are caused by occupational accidents or work-related diseases.^1^ In 2019, the U.S. Bureau of Labor Statistics reported 5,333 work-related fatal accidents.^2^ The U.S. National Institute of Occupational Safety and Health (NIOSH) mentions that healthcare workers are more exposed to infectious respiratory diseases such as tuberculosis^3^ and that they would be the most affected by a possible public health emergency, as occurred in the current COVID-19 pandemic.^4^

Risk perception is a subjective assessment that measures the probability of experiencing an accident or a disease due to exposure to a source of risk^5^; moreover, it has the potential of shaping health-related behaviors, reducing exposure and prioritizing protection measures.^6^ Proper assessment of risk perception would allow for the formulation of risk policies based on knowledge of associated factors.^7^ Several studies have shown that high risk perception levels are related to lower rates of occupational accidents.^8,9^

Risk perception is a highly personal process of decision making, based on individual’s frame of reference developed over a lifetime, among many other factors^10^; in this sense, it is essential that workers properly evaluate the risks around them to make relevant decisions regarding prevention of these risks, because worker’s occupational risk perception determines accurate or inaccurate risk assessment, which can cause an accident or disease to occur.^11^

Knowledge on the factors associated with occupational risk perception could help the health personnel and health centers to take relevant precautions and measures for a safe work placement. In Peru, evidence on the matter is scarce, and the studies that evaluated occupational risk in the health personnel were usually conducted in small, non-probabilistic samples from the hospital setting. Therefore, the aim of the present study is to determine the levels of occupational risk perception and its associated factors in physicians and nurses of health facilities in Peru during 2016, through a nationally representative sample.

## METHODS

### DESIGN

An analytical cross-sectional study was conducted with secondary data from the National Survey of Health Users Satisfaction (Encuesta Nacional de Satisfacción de Usuarios en Salud, ENSUSALUD) 2016. The study population consisted of nurses and physicians who were working at health facilities in all departments of Peru, from May to June 2016.

The study project was approved by the Research Ethics Institutional Committee of Universidad Científica del Sur (code: 499-2020-PRE15). Data from the ENSUSALUD 2016 do not contain information that identifies participants. Database is available at the following link: https://bit.ly/susalud2016.

### DATA SOURCE

The ENSUSALUD 2016 was conducted by the National Health Authority (Superintendencia Nacional de Salud, SUSALUD), and the research question was answered using the questionnaire 2, which aimed to determine of physicians’ and nurses’ perception about the health facility where they work. The ENSUSALUD 2016 included facilities administered by the Ministry of Health and regional governments (MH-RG), the Social Health Security (EsSalud), the Armed Forces and the Peruvian National Police (AF and PNP) health insurances, and private clinics. The sample comprised 5,098 professionals from 183 health facilities throughout Peru. Sampling employed a two-stage probabilistic approach, stratified and independent in each department. Health facilities were selected at the first stage, and nurses and physicians at the second one.

The present analysis included records from nurses and physicians aged above 18 years old of both sexes and with Peruvian nationality. Records of professionals older than 65 years of age were excluded, since they were above retirement age in Peru, as well as records with incomplete or inconsistent data.

### VARIABLES

The problem variable of was occupational risk perception, which was assessed by two questions: 1) «Indicate how often the following happens: Exposure to people with very contagious diseases?» 2) «Indicate how often the following happens: Exposure or contact with substances that could affect your health?». In both questions, the Likert-scale answer options were: never = 1, hardly ever = 2, occasionally = 3, almost always = 4, and always = 5. Scores for the two questions were added and divided by three, in order to determine the following categories: low risk (2 to 4 points), moderate risk (5 to 7 points), and high risk (8 to 10 points).

Sociodemographic variables, such as age (years), sex (male, female), marital status (married/cohabitating, widowed/divorced, single), family cohabitation «currently, do you live with your family?» (yes, no), monthly income in Peruvian soles (<3,000, 3,000-5,000, >5,000) were included as possible associated variables.

Furthermore, the analysis included other variables that, according to previous studies, would be related to the occurrence of occupational accidents or diseases in the health personnel, such as institution of origin (MH-RG, EsSalud, AF and PNP health insurances, private clinics),^12^ years working at the health facility,^13^ weekly working hours at the health facility, having a specialty (yes, no),^14^ suffering from a chronic disease (yes, no),^15^ previous occupational accident (yes, no), physical violence in the health facility in the last 12 months (no, yes),^16^ receiving occupational risk training (no, yes),^17^ receiving protection equipment (no, yes).^18^

### STATISTICAL ANALYSIS

The database was discharged from the SUSALUD website (https://bit.ly/susalud2016) and was imported to and analyzed by Stata/MP statistical software, version 16 (Stata Corporation, College Station, Texas, USA). Categorical variables were expressed as frequencies and weighted percentages (using a complex sampling method). Numerical variables were expressed as mean and standard error, according to the normality of their distribution. Differences between the professions (nurses and physicians) and levels of occupational risk perception (low, moderate, and high) were assessed using the Wald test and the chi-square test corrected for survey design. Identification of the factors associated with occupational risk perception was performed using ordinal logistic regression, obtaining *j* (IC95%), after testing for the proportional hazard assumption through the *gologit2* command with *autofit* option, which supports survey design.^19^ Two models were developed, one crude and another adjusted, which included variables that resulted associated in the crude model (p < 0.05). Moreover, the adjusted models evaluated the presence of multicollinearity through manual calculation of variance inflation factor (VIF). All calculations were performed considering the ENSUSALUD 2016 complex sampling, through the *svy* commands of Stata.

## RESULTS

**Figure 1:**
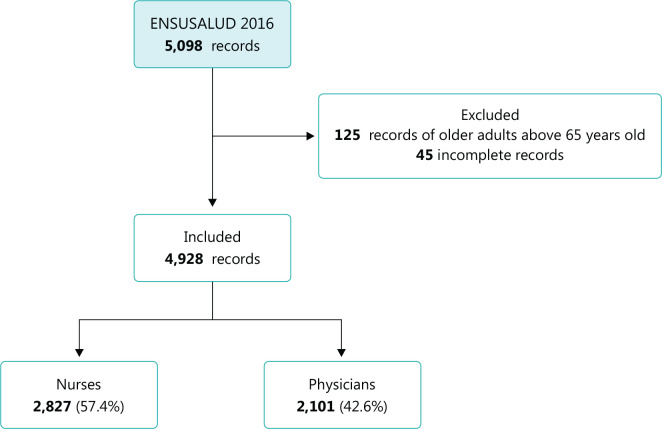
Flowchart of study participant’s selection. Nurses and physicians in Peru, 2016. ENSUSALUD 2016 = National Survey of Health Users Satisfaction (Encuesta Nacional de Satisfacción de Usuarios en Salud) 2016.

Of the 5,098 records obtained in the sample, 125 were excluded because they belonged to professionals older than 65 years of age, and 45 because they had incomplete data for the variable «monthly income». Therefore, 4,928 records remained in the final analysis, of which 59.1% (n = 2,827) were from nurses, and 40.9% (n = 2,101) from physicians (Figure 1).

**Figure 2:**
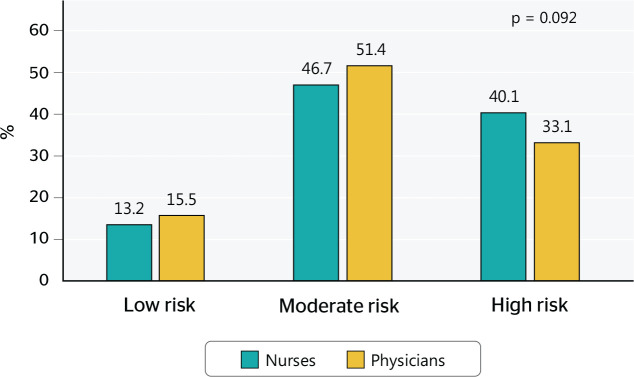
Levels of occupational risk perception among nurses and physicians in Peru, 2016. Percentages are weighted according to the National Survey of Health Users Satisfaction 2016 complex sampling.

Significant differences between nurses and physicians were observed regarding age and sex. Mean age of physicians was higher than that of nurses. There was a higher proportion of female nurses (93.3%) and a lower proportion of female physicians (31.2%). On average, nurses had more years working at the health facility compared to physicians, and a higher percentage of them lived with their family, received occupational risk prevention or biosafety training, and were provided with protective equipment by their institution. On average, physicians worked more weekly hours, and most of them earned a higher income, had a chronic disease, and were physically assaulted at the health facility more often, compared to nurses (Table 1). The levels of occupational risk perception were similar between nurses and physicians (p = 0.092), with slight variations favoring physicians when risk perception was low or moderate, and favoring nurses when risk perception was high (Figure 2).

**Table 1:** Characteristics of nurses and physicians in Peru, 2016

Characteristic	Nurses (n = 2,827)	Physicians (n = 2,101)	p-value†
	n (%)	n (%)	
Age, mean (SE)	42.8 (0.41)	44.4 (0.54)	0.017‡
Sex			<0.001
Male	238 (6.7)	1.569 (68.8)	
Female	2,589 (93.3)	532 (31.2)	
Marital status			0.809
Married/cohabitating	1,808 (62.6)	1,455 (64.5)	
Widowed/divorced	200 (5.4)	117 (5.1)	
Single	819 (32.0)	529 (30.4)	
Institution of origin			0.284
MH-RG	1,347 (47.7)	962 (43.0)	
EsSalud	1,236 (27.7)	981 (34.4)	
AF and PNP	62 (9.9)	33 (8.4)	
Private clinics	182 (14.7)	125 (14.2)	
Years working at the HF, mean (SE)	10.5 (0.36)	8.4 (0.38)	<0.001‡
Weekly working hours at the HF, mean (SE)	41.0 (0.32)	52.8 (0.69)	<0.001‡
Monthly income (PEN)^*^			<0.001
<3,000	1,412 (59.4)	70 (4.3)	
3,000-5,000	1,241 (33.4)	666 (29.7)	
>5,000	174 (7.2)	1,365 (66.0)	
Specialty			0.664
Yes	1,936 (65.2)	1,555 (63.7)	
No	891 (34.8)	546 (36.3)	
Family cohabitation			<0.001
Yes	2,571 (90.4)	1,681 (83.4)	
No	256 (9.6)	416 (16.6)	
Chronic disease			0.003
No	2,107 (78.3)	1,548 (69.7)	
Yes	720 (21.7)	553 (30.3)	
Occupational accident			0.242
No	2,464 (88.2)	1,924 (90.7)	
Yes	363 (11.8)	177 (9.3)	
Physical violence in the HF			0.020
No	1,770 (61.5)	1,110 (53.8)	
Yes	1,057 (38.5)	991 (46.2)	
Received occupational risk training			<0.001
No	1,401 (49.9)	1,462 (71.4)	
Yes	1,426 (50.1)	639 (28.6)	
Received protective equipment			<0.001
No	997 (37.9)	1,071 (52.4)	
Yes	1,830 (62.1)	1,030 (47.6)	

* 1 PEN = 0.30 USD in May 2016.

† Chi-square test corrected for survey design.

‡ Wald test.

AF and PNP = Armed Forces and Peruvian National Police health insurances; EsSalud = Social Health Insurance; HF = health facility; MH-RG = Ministry of Health and regional governments; SE = standard error.

The bivariate analysis of the nurse group found significant differences in the level of occupational risk regarding institution of origin (p = 0.035), mean weekly working hours (p < 0.001), physical violence in the health facility (p = 0.008), and receiving protective equipment (p = 0.001) (Table 2). The bivariate analysis of the physician group found significant differences in the level of occupational risk regarding presence of chronic disease (p = 0.020) and receiving occupational risk training (p = 0.042) (Table 3).

**Table 2:** Differences according to categories of occupational risk perception among nurses in Peru, 2016

Characteristic	Occupational risk perception			p-value
	Low (n = 331)	Moderate (n = 1,194)	High (n = 1,302)	
	n (%)	n (%)	n (%)	
Age, mean (SE)	44 (1.37)	42 (0.58)	42 (0.61)	0.106‡
Sex				0.098
Male	22 (13.4)	93 (35.9)	123 (50.7)	
Female	309 (13.1)	1,101 (47.5)	1,179 (39.4)	
Marital status				0.370
Married/cohabitating	209 (14.1)	768 (48.0)	831 (37.9)	
Widowed/divorced	22 (6.9)	74 (48.5)	104 (44.6)	
Single	100 (12.4)	352 (43.7)	367 (43.9)	
Institution of origin				0.035
MH-RG	183 (14.2)	568 (45.9)	596 (39.9)	
EsSalud	110 (8.5)	516 (44.8)	610 (46.7)	
AF and PNP	17 (24.3)	33 (56.9)	12 (18.8)	
Private clinics	21 (11.2)	77 (45.7)	84 (43.1)	
Years working at the HF, mean (SE)	10.6 (1.0)	10.5 (0.5)	10.4 (0.5)	0.815‡
Weekly working hours at the HF, mean (SE)	38.6 (0.44)	40.9 (0.47)	41.9 (0.53)	<0.00^1‡^
Monthly income (PEN)^*^				0.369
<3,000	206 (15.6)	588 (46.7)	618 (37.7)	
3,000-5,000	109 (10.4)	532 (46.5)	600 (43.1)	
>5,000	16 (6.2)	74 (47.2)	84 (46.6)	
Specialty				0.870
Yes	192 (13.3)	816 (47.3)	928 (39.4)	
No	139 (12.8)	378 (45.5)	374 (41.7)	
Family cohabitation				0.518
Yes	296 (13.4)	1,093 (47.0)	1,182 (39.6)	
No	35 (10.9)	101 (43.2)	120 (45.9)	
Chronic disease				0.264
No	239 (12.8)	875 (45.5)	993 (41.7)	
Yes	92 (14.6)	319 (50.9)	309 (34.5)	
Occupational accident				0.054
No	301 (13.9)	1,057 (47.4)	1,106 (38.7)	
Yes	30 (7.9)	137 (41.2)	196 (50.9)	
Physical violence in the HF				0.008
No	236 (16.4)	764 (44.0)	770 (39.6)	
Yes	95 (8.0)	430 (50.9)	532 (41.1)	
Received training in occupational risks				0.397
No	192 (15.1)	621 (46.8)	588 (38.1)	
Yes	139 (11.3)	573 (46.5)	714 (42.2)	
Received protective equipment				0.001
No	146 (18.1)	455 (50.7)	396 (31.2)	
Yes	185 (10.2)	739 (44.2)	906 (45.6)	

* 1 PEN = 0.30 USD in May 2016.

† Chi-square test corrected for survey design.

‡ Wald test.

AFF and PNP = Armed Forces and Peruvian National Police health insurances; EsSalud = Social Health Insurance; HF = health facilities; MH-RG = Ministry of Health and regional governments; SE = standard error.

**Table 3:** Differences according to categories of occupational risk perception among physicians in Peru, 2016

Characteristic	Occupational risk perception			p-value†
	Low (n=264)	Moderate (n=975)	High (n=898)	
	n (%)	n (%)	n (%)	
Age, mean (SE)	46.7 (1.4)	44.3 (0.9)	43.5 (0.6)	0.106‡
Sex				0.356
Male	186 (13.7)	719 (51.8)	664 (34.5)	
Female	70 (19.5)	243 (50.6)	219 (29.9)	
Marital status				0.365
Married/cohabitating	191 (16.9)	677 (51.6)	587 (31.5)	
Widowed/divorced	14 (15.5)	51 (35.7)	52 (48.8)	
Single	51 (12.5)	234 (53.8)	244 (33.7)	
Institution of origin				0.102
MH-RG	113 (10.5)	451 (49.2)	398 (40.3)	
EsSalud	110 (18.3)	432 (50.2)	439 (31.5)	
AF and PNP	6 (19.7)	19 (66.6)	8 (13.7)	
Private clinics	27 (21.4)	60 (52.2)	38 (26.4)	
Years of working at the HF, mean (SE)	9.9 (1.0)	8.1 (0.5)	8.3 (0.5)	0.278‡
Weekly working hours at the HF, mean (SE)	49.5 (2.1)	53.9 (0.9)	52.6 (1.1)	0.379‡
Monthly income (PEN)^*^				0.542
<3,000	8 (12.0)	34 (62.4)	28 (25.6)	
3,000-5,000	72 (18.2)	295 (46.7)	299 (35.1)	
>5,000	176 (14.5)	633 (52.9)	556 (32.6)	
Specialty				0.103
Yes	185 (12.6)	703 (51.8)	667 (35.6)	
No	71 (20.6)	259 (50.9)	216 (28.5)	
Family cohabitation				0.188
Yes	219 (16.0)	770 (52.8)	696 (31.2)	
No	37 (13.2)	192 (44.5)	187 (42.3)	
Chronic disease				0.020
No	184 (12.8)	711 (50.4)	653 (36.8)	
Yes	72 (21.7)	251 (53.9)	230 (24.4)	
Occupational accident				0.749
No	237 (15.3)	894 (52.1)	793 (32.6)	
Yes	19 (17.7)	68 (44.8)	90 (37.5)	
Physical violence in the HF				0.224
No	142 (15.8)	506 (47.7)	462 (36.5)	
Yes	114 (15.2)	456 (55.8)	421 (29.0)	
Received occupational risk training				0.042
No	200 (17.9)	694 (51.6)	568 (30.5)	
Yes	56 (9.5)	268 (51.1)	315 (39.4)	
Received protective equipment				0.220
No	167 (17.3)	507 (53.4)	397 (29.3)	
Yes	89 (13.5)	455 (49.3)	486 (37.2)	

* 1 PEN = 0.30 USD in May 2016.

† Chi-square test corrected for survey design.

‡ Wald test.

AFF and PNP = Armed Forces and Peruvian National Police health insurances; EsSalud = Social Health Insurance; HF = health facilities; MH-RG = Ministry of Health and regional governments; SE = standard error.

The crude model for the nurse group revealed that institution of origin (specifically the category AF and PNP health insurances), weekly working hours, occurrence of occupational accident, and receiving protective equipment were found to be associated with higher perceived occupational risk. These associations were maintained in the adjusted model, except for institution of origin. Nurses who worked more hours per week, who suffered an occupational accident, and who received protective equipment were more likely to have a higher perceived occupational risk (Table 4).

**Table 4:** Multivariate analysis to determine the factors associated with occupational risk perception among nurses and physicians in Peru, 2016

Characteristic	Nurses		Physicians	
	Crude model	Adjusted model	Crude model	Adjusted model‡
	OR (95%CI)	OR (95%CI)	OR (95%CI)	OR (95CI%)
Age	0.99 (0.97-1.01)		0.98 (0.97-0.99)	0.98 (0.97-0.99)
Sex				
Male	1	-	1	-
Female	0.69 (0.43-1.11)	-	0.75 (0.49-1.14)	-
Marital status				
Married/cohabitating	1	-	1	-
Widowed/divorced	1.43 (0.95-2.15)	-	1.84 (0.79-4.27)	-
Single	1.25 (0.87-1.81)	-	0.80 (0.81-1.76)	-
Institution of origin				
MH-RG	1	1	1	1
EsSalud	1.40 (0.96-2.05)	1.27 (0.87-1.86)	0.62 (0.41-0.94)	0.59 (0.39-0.90)
AF and PNP	0.42 (0.21-0.86)	0.50 (0.24-1.01)	0.36 (0.16-0.81)	0.33 (0.14-0.72)
Private clinics	1.18 (0.80-1.74)	0.93 (0.61-1.41)	0.31 (0.31-0.77)	0.42 (0.27-0.66)
Year working at the HF	0.99 (0.98-1.01)	-	0.99 (0.97-1.01)	-
Weekly working hours at the HF	1.02 (1.01-1.03)	1.01 (1.01-1.03)	1.01 (0.99-1.01)	-
Monthly income (PEN)^*^				
<3,000	1		1	-
3,000-5,000	1.32 (0.96-1.82)	-	1.14 (0.62-2.09)	-
>5,000	1.60 (0.68-3.81)	-	1.16 (0.69-1.93)	-
Specialty				
Yes	1	-	1	1
No	1.09 (0.77-1.54)	-	0.66 (0.44-0.99)	0.66 (0.44-0.99)
Family cohabitation				
Yes	1	-	1	-
No	1.29 (0.82-2.03)	-	1.52 (0.94-2.46)	-
Chronic disease				
No	1	-	1	1
Yes	0.77 (0.56-1.05)	-	0.54 (0.36-0.81)	0.65 (0.43-0.97)
Previous occupational accident				
No	1	1	1	-
Yes	1.67 (1.08-2.59)	1.59 (1.05-2.41)	1.10 (0.51-2.40)	-
Physical violence in the HF				
No	1	-	1	-
Yes	1.26 (0.92-1.71)	-	0.80 (0.57-1.14)	-
Received occupational risk training				
No	1	-	1	1
Yes	1.23 (0.89-1.70)	-	1.61 (1.12-2.30)	1.78 (1.22-2.60)
Received protective equipment				
No	1	1	1	-
Yes	1.88 (1.35-2.62)	1.65 (1.18-2.32)	1.41 (0.98-2.02)	-

* 1 PEN = 0.30 USD in May 2016.

† Adjusted for institution of origin, weekly working hours at the health facility, previous occupational accident, and receiving protective equipment.

‡ Adjusted for age, institution of origin, having a specialty, chronic disease, and receiving occupational risk training.

AFF and PNP = Armed Forces and Peruvian National Police health insurances; EsSalud = Social Health Insurance; HF = health facilities; MH-RG = Ministry of Health and regional governments; OR: odds ratio; SE = standard error.

The crude model for the physician group revealed that age, institution of origin, having a specialty, suffering from a chronic disease, and receiving training were found to be associated with higher perceived occupational risk. All these associations remained significant in the adjusted analysis. Physicians who worked at facilities affiliated with the social health insurance, AF and PNP social insurances, and private clinics were less likely to have a higher perceived occupational risk, compared to physicians who worked at affiliated with the MH-RG. Furthermore, physicians who did not have a specialty and who suffered from a chronic disease were less likely to have a higher perceived occupational risk. Conversely, physicians who received protective equipment were more likely to have a higher perceived occupational risk (Table 4).

The proportional hazard assumption was confirmed for the nurse group (F = 0.32, p = 0.902) and the physician group (F = 0.55, p = 0.795), which supports the use of ordinal logistic regression. The adjusted models did not show evidence of multicollinearity in any of the variables (VIF ≈ 1).

## DISCUSSION

The levels of occupational risk perception were similar between nurses and physicians. Weekly working hours, occurrence of a previous occupational accident, and receiving protective equipment showed to be associated with a higher perceived occupational risk in nurses. Institution of origin, having a specialty, suffering from a chronic disease, and receiving occupational risk training showed to be associated with higher perceived occupational risk in physicians.

## INTERPRETATION OF RESULTS

Physicians who worked at social health insurance or private facilities were less likely to perceive occupational risk than those who worked at facilities affiliated with the MH. The Peruvian health system is fragmented into a public sector, a social sector, and a private sector, which in turn are divided into several types of insurances and allowances. This disarticulation affects the efficiency and equity of services.^20^ Perhaps physicians perceived that the greatest resources, in terms of infrastructure and equipment, are found in the social health insurance and private facilities, in contrast to the budget deficiencies and limitations observed in public health facilities, particularly when 70% of workers rely on the MH.^21^ Therefore, physicians take lower occupational risks in facilities with greater resources.

Physicians with no specialty were less likely to have higher perceived occupational risk. This finding probably results from the fact that these professionals work mostly at primary care facilities or perform administrative functions, which would imply lower exposure to occupational risks and thus lower perceived risk. This physicians’ security or confidence regarding their workplace has been described as a factor that could predict occupational risk,^22^ showing that the greater the confidence, the lower risk perception.^23^

Physicians with chronic diseases were less likely to perceive occupational risk. Chronic diseases are risk factors that could increase vulnerability in the health personnel, because these diseases can be complicated by in-hospital infections; therefore, a higher perceived risk would be expected, similar to what proposed for the general population facing epidemic events.^24^ However, risk perception was lower in both groups, but the difference was significant only in physicians, which could be explained by the way these professionals cope with their own chronic diseases. In this respect, it has been described that the prevalences of chronic disease in physicians are underestimated, since these professionals do not recognize their diseases and just «accept, adapt, and carry on» with their illness, or sometimes «carry on and adapt» before accepting their illness,^25^ avoiding recognize the problem. These personality traits can affect the diagnosis and treatment of chronic disease; therefore, the level of perceived risk is expected to be higher.

Nurses who have suffered an occupational accident were more likely to have a higher perceived occupational risk. Healthcare professionals who had experienced an occupational accident inside the health facility may be more aware of the risk and thus, maintain a higher level of occupational risk perception, as reported in a study of nurses in Spain, which observed a higher perceived occupational risk in those who had a previous occupational accident.^26^

Physicians who received training were more likely to have a higher perceived occupational risk. This probably results from the greater level of knowledge these professionals have about occupation safety and health.^27^ A positive correlation has been identified between systematic knowledge obtained to gather relevant information, risk perception levels, and health-related behaviors.^28^ Training sessions conducted with urgency to combat infectious diseases, such as in the current COVID-19 pandemic, result beneficial for a better patient management and protection of the health personnel,^29^ which, according to our findings, also increase their risk perception, and possibly contributed to reduce the number of occupational accidents.

Nurses who received protective equipment presented a higher perceived occupational risk. Evidence suggests that the use of protective equipment classified as important health resources could reduce risk perception.^23^ A study that assessed the factors associated with use of protective equipment in Italian physicians during the COVID-19 pandemic found that those who had access to this equipment were less likely to have a higher perceived risk.^30^ However, it should be acknowledged that this study was conducted at the beginning of the pandemic, when knowledge about the route of infection was limited, thus resulting in a lower perceived risk. However, in the present study this variable had an opposite behavior. Knowledge and adequacy obtained over time could probably explain this finding.

### LIMITATIONS AND STRENGTHS

The present study presents the following limitations: first, the answers obtained to measure occupational risk perception may be subjected to social desirability and recall bias, which could increase final prevalences. Second, measurement of risk perception was not based on a validated scale; therefore, comparisons with similar studies should be undertaken with caution. Third, some variables that could explain occupational risk perception and could be associated factors may not be available in the database analyzed. Fourth, due to the cross-sectional design of this study, it was not possible to establish a causal relationship between occupational risk perception and its associated factors. As a strength it should be acknowledged that these data allow generalizing the results for the entire population of nurses and physicians in Peru.

## CONCLUSIONS

The levels of occupational risk perception were similar between nurses and physicians in Peru. However, the associated factors differed between the two groups of professionals. It is necessary to conduct more studies to assess occupational risk perception in healthcare professionals using validated instruments, in order to obtain more reliable and accurate results. Furthermore, it is suggested that the associated factors found in the present study should be taken into account when formulating norms or laws related to occupational safety of health personnel.
